# Regression Equation for Predicting Source Strength Detection Rate in Iodine-125 Seed Brachytherapy

**DOI:** 10.7759/cureus.107495

**Published:** 2026-04-21

**Authors:** Masato Takanashi, Isao Kuroda, Tatsuhiko Zama, Yoshiaki Katada, Masataka Hoshina, Masaya Noguchi, Koichi Masuda

**Affiliations:** 1 Department of Radiology and Radiation Oncology, Tokyo Medical University Ibaraki Medical Center, Ami, JPN; 2 Department of Urology, Tokyo Medical University Ibaraki Medical Center, Ami, JPN

**Keywords:** 125i seed brachytherapy, batch assay, dead seed, iodine-125, prostate cancer, single-seed assay, well-type ionization chamber

## Abstract

Prostate cancer is one of the most common malignant tumors in males, and its incidence is increasing worldwide. In Japan, iodine-125 (125I) seed brachytherapy was introduced in 2003 and has since been used to treat many patients. Iodine-125 (125I) seed brachytherapy is a treatment in which multiple small capsules containing 125I are implanted into the prostate. Naturally, if the activity of the 125I source differs from the manufacturer’s stated value, it may affect treatment outcomes. European and American guidelines state that it is the responsibility of medical physicists to verify the source activity before treating patients. In previous studies, there have been three cases in Japan where seeds with incorrect source activity were delivered to medical institutions since 2017. Specifically, seeds with source activity different from what the institutions had ordered, or dead seeds, were delivered. This has led to a loss of trust in the source activity values published by manufacturers. An investigation conducted in response to these incidents revealed that only 16% of facilities verified source activity, and only 52% verified the number of seeds. Reasons cited for the low verification rates included a lack of necessary equipment, insufficient knowledge of verification procedures, a shortage of staff capable of performing verification, and the fact that many seeds distributed in Japan cannot be re-sterilized. The American Association of Physicists in Medicine (AAPM) advocates for the importance of source strength verification at user facilities. Since we began 125I seed brachytherapy, our facility has continuously performed source strength verification on all delivered blister packs. The single-seed assay recommended by the AAPM is difficult to perform in Japan. As an alternative method, a batch assay using an ionization chamber or a well-type ionization chamber to measure multiple seeds simultaneously has been proposed. At our facility, we calculate the source intensity detection rate from measurements using a well-type ionization chamber to verify the accuracy of the source strength. Our facility uses AgX100 (Buford, GA: Theragenics Corporation) as the source and the CRC-15R well-type ionization chamber (Ramsey, NJ: Capintec Corp.) as the measuring instrument. While there are scattered reports verifying detection rates in batch assays as prior research, there are very few papers discussing regression equations relating the number of sources to detection rates. The objective of this study is to validate a regression equation for predicting appropriate detection rates for each number of radiation sources based on accumulated data. Microsoft Office - Excel 2019 (Redmond, WA: Microsoft Corp.) was used to calculate the regression equation. The guidelines explain how to respond when batch assay results deviate from nominal values. Specific control limits, such as acceptance limits and intervention limits, are indicated. In seed therapy, the insertion of dead seeds or sources with mismatched calibration dates can have significant consequences; therefore, source strength measurement is a critical verification procedure in this treatment modality. We believe the regression equation derived from this study will serve as a useful reference for facilities that have already started 125I seed brachytherapy, as well as those planning to do so in the future.

## Introduction

Prostate cancer is one of the most common malignant tumors in males, and its incidence is increasing worldwide [1,2]. In Japan, Iodine-125 (125I) seed brachytherapy was introduced in 2003 and has been used to treat many patients [3]. One of the advantages of 125I seed brachytherapy is the low incidence of reported adverse events [4,5]. Recent studies also suggest that 125I seed brachytherapy is safe and has potential for further development [6]. Iodine-125 (125I) seed brachytherapy is a treatment in which multiple small capsules containing radioactive 125I are implanted into the prostate. Naturally, if the source strength of 125I differs from the manufacturer’s nominal value, it may affect treatment outcomes. European and American guidelines state that it is the responsibility of medical physicists to verify source activity before treating patients [7-12]. In Japan, however, few facilities perform such verification. Reasons for this include a lack of verification equipment, insufficient knowledge of verification procedures, a shortage of personnel to perform verification, and the inability to re-sterilize many of the sources currently in circulation in the country. For these reasons, it is difficult to perform single-seed assays domestically [13,14]. Consequently, batch assays, which allow for the measurement of multiple sources under sterile conditions, have been proposed as an alternative to single-seed assays. The guidelines specify that the tolerance limit for batch assays of all delivered radiation sources is 3%, and the intervention limit is 5%. To date, studies on batch assays have been scattered [15-19]. At our facility, we calculate the source intensity detection rate based on measurements using a well-type ionization chamber to verify source intensity. Our facility uses AgX100 (Buford, GA: Theragenics Corporation) as the source and the CRC-15R well-type ionization chamber (Ramsey, NJ: Capintec Corp.) as the measuring instrument. We have evaluated and reported the normality of the detection rates based on the data accumulated to date [20]. We have also reported clinical cases involving blister packs that exceeded permissible limits [21].

## Materials and methods

Study design

**Figure 1:**
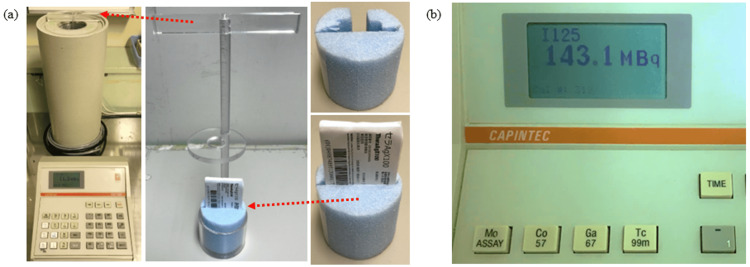
Method for measuring radiation source strength using a well-type ionization chamber (batch assay at our facility). At our facility, we verify source strength using a batch assay with a well-type ionization chamber. The well-type ionization chamber we use is the CRC-15R (Ramsey, NJ: Capintec Corp.). As a measurement procedure, we fix the blister pack to a homemade Styrofoam pedestal, as shown in image (a). Next, it is attached to an acrylic hanging rod and placed in the well-type ionization chamber. The measured values are then read, as shown in image (b).

At our facility, we verify source intensities using batch assays with a well-type ionization chamber. The well-type ionization chamber used is the CRC-15R. As shown in Figure 1a, the measurement procedure begins by securing the blister pack to a custom-made Styrofoam block. Next, the blister pack is attached to an acrylic suspension rod and placed inside the well-type ionization chamber. The measurement values are then read as shown in Figure 1b.

The half-life of 125I is 59.4 days. In clinical settings, it is not always possible to perform measurements on the specified date and time. Therefore, when the measurement date differs from the test date, a decay correction was applied to the measured values. Additionally, in accordance with recommendations from the International Atomic Energy Agency, Japan amended the Act on the Regulation of Radioactive Isotopes and Related Matters on October 1, 2023, to ensure the reliability of radiation measurements. This amendment mandates that “inspections and calibrations of radiation measurement equipment be appropriately combined and conducted annually.” Compared to the regulations prior to the amendment, this law requires the use of regularly calibrated dosimeters. The dosimeters used in this study were also regularly calibrated, ensuring the reliability of the measurement values. The radiation source used in this study was the AgX100. Three types of source strengths - 11.0 MBq, 13.1 MBq, and 15.3 MBq - are commercially available; however, this study focused on the 11.0 MBq model, which has been the most frequently delivered to our hospital to date. Our hospital received a total of 433 blister packs during the study period from January 2020 to May 2023. The breakdown is shown in Table 1.

**Table 1:** Our facility's delivery record by number of packaged radiation sources. The table shows the actual deliveries of blister packs by number of radiation sources at our facility from January 2020 to May 2023. As can be seen from the table, the proportions of five- and 20-count blister packs are significant. For the AgX100, the number of orders for blister packs containing one to four sources is lower. This is because, based on the manufacturer’s ordering rules, orders for blister packs containing one to four sources are limited to one per order. Specifically, for example, when ordering 74 sources, the breakdown would be three sets of 20 source packs, two sets of five source packs, and one set of four source packs.

Number of packed radiation sources	1	2	3	4	5	20
Number of measurements blister packs (time)	20	15	18	30	197	153
Ratio to the total number of packs received (%)	4.6	3.5	4.2	6.9	45.5	35.3

**Figure 2:**
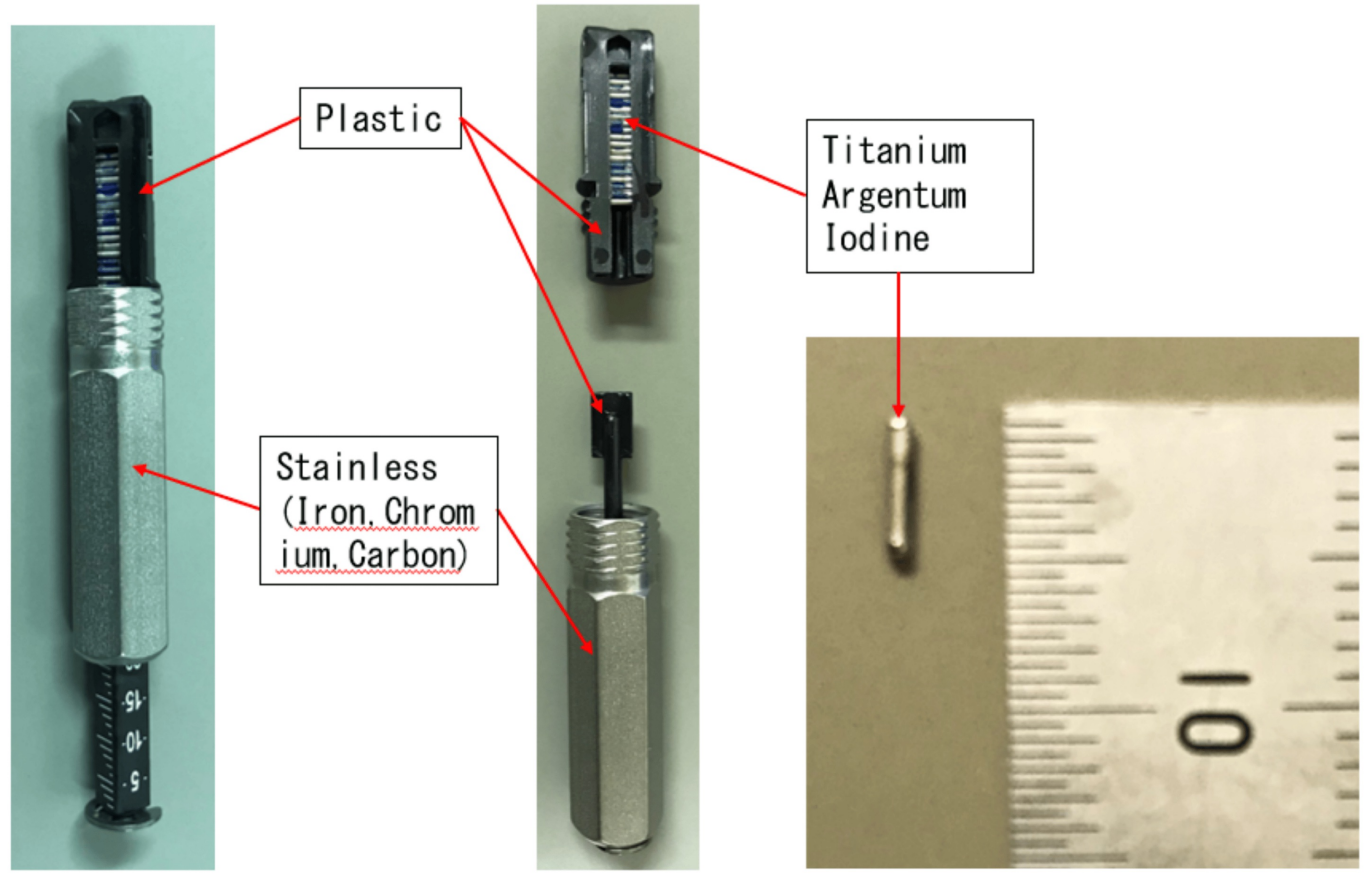
Cartridge and source materials. This image shows the materials constituting the cartridge enclosed in the blister pack. The cartridge is composed of plastic and stainless steel, while the radiation source consists of titanium, silver, and iodine.

**Figure 3:**
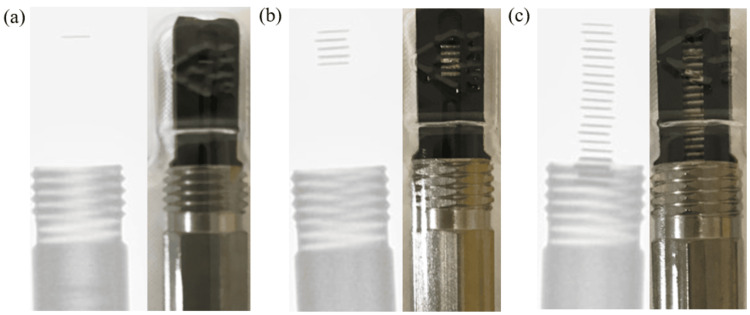
X-ray fluoroscopy image of a blister pack. This image shows X-ray fluoroscopic images of blister packs containing (a) one, (b) five, and (c) 20 radiation sources. As can be seen in the figure, the position of the radiation sources varies depending on the number of sources. As shown in (a) and (b), when there are one or five sources, all radiation sources are located within the plastic portion. On the other hand, as shown in (c), when there are 20 sources, some of the radiation sources are located inside the stainless steel portion.

**Figure 4:**
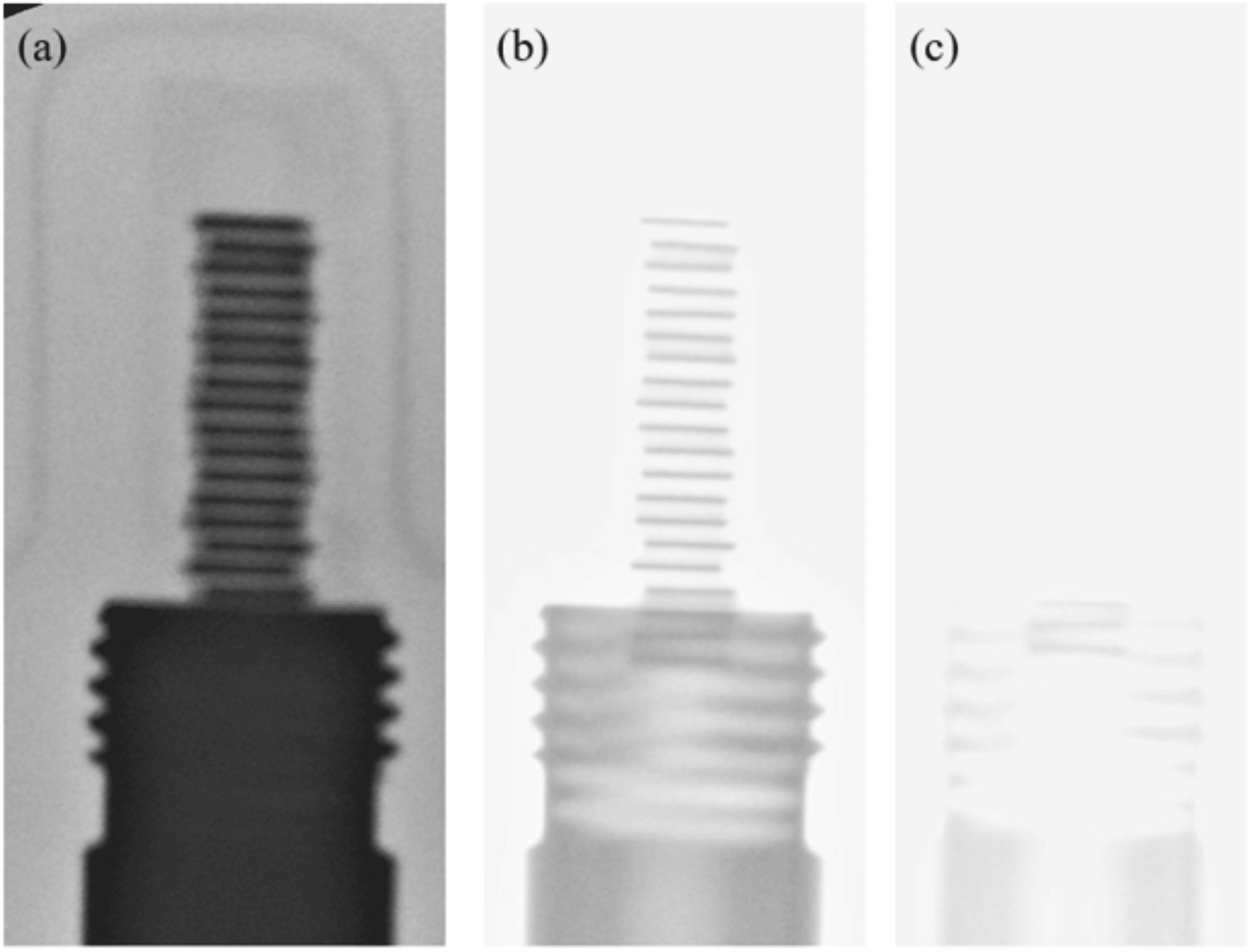
Differences in the appearance of blister packs under different X-ray fluoroscopy parameters. The X-ray fluoroscopy conditions are as follows: (a) 44 kV, 0.4 mA; (b) 65 kV, 0.5 mA; and (c) 75 kV, 0.9 mA. In (a), it can be seen that the individual sources are densely arranged. However, the sources inside the stainless steel are obscured by the shielding and cannot be clearly identified. In (b), the sources both inside and outside the stainless steel can be clearly identified. However, the sources inside the stainless steel overlap with the screw threads and cannot be clearly identified. In (c), compared to (a) and (b), the higher voltage photons increase penetration, allowing the sources inside the stainless steel to be clearly visualized. From the above, it can be seen that there are approximately three sources located inside the stainless steel.

Figure 2 shows the materials used to construct the cartridge enclosed in the blister pack. The cartridge is made of plastic and stainless steel, and the radiation source consists of titanium, silver, and iodine. Figures 3a-3c show X-ray fluoroscopic images of blister packs containing (a) one, (b) five, and (c) 20 radiation sources. As can be seen from the figures, the position of the sources varies depending on the number of sources. In the case of one or five sources, all sources are located within the plastic portion. On the other hand, as shown in Figures 3a-3c, 4a-4c, for 20 sources, approximately three sources are located within the stainless steel portion. Next, Figures 4a-4c show how the appearance of the blister pack varies depending on the X-ray fluoroscopy settings.

To ensure measurement stability, readings were taken 60 s after inserting the radiation source into the well-type ionization chamber. The formula for calculating the detection rate is as follows:

$\text{Detection rate (\%)} = \left( \frac{ \text{Measured value by well-type ionization chamber (MBq)} }{ \text{Manufacturer's nominal value (MBq)} \times \text{Number of packaged sources in blister pack (units)} } \right) \times 100$

Statistical analysis

To derive the regression equation, we plotted the number of packaging radiation sources on the x-axis and the detection rate on the y-axis, then used Microsoft Office - Excel 2019 (Redmond, WA: Microsoft Corp.) to fit a regression line.

## Results

**Figure 5:**
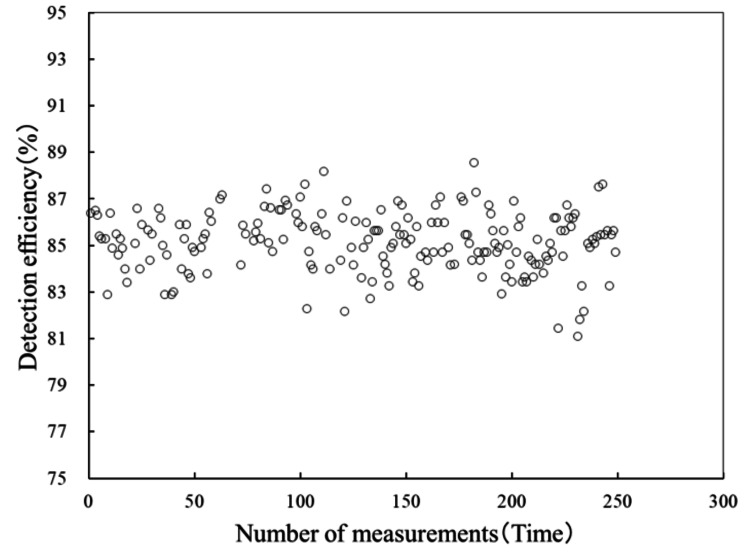
Regression formula for detection rate of one to five packing radiation sources. The graph shows a scatter plot of the detection rates for five-seed blister packs at this facility from January 2020 to May 2023. Since the control range for five-seed blister packs is 80.9-89.4%, it can be seen that all blister packs fall within this range.

**Figure 6:**
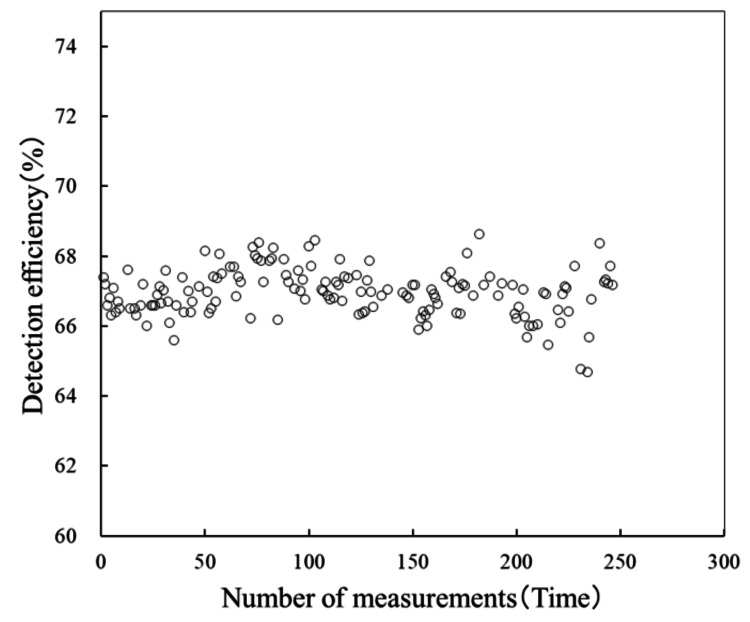
Regression formula for detection rate of one to 20 packing radiation sources. The graph shows a scatter plot of the detection rates for 20-count blister packs at this facility from January 2020 to May 2023. Since the acceptable range for 20-count blister packs is 63.6-70.3%, it can be seen that all blister packs fall within this range.

Table 2 shows the differences in tolerance range by the number of packaged radiation sources and the detection rate per source. Next, Figures 5, 6 show scatter plots of the detection rates for five-blister packs and 20-blister packs at this facility from January 2020 to May 2023.

**Table 2:** Differences in detection range by the number of sources in a packaging line and the detection rate per source. ※2/※1 Detection rate per radiation source: this figure was calculated by dividing the average cumulative detection rate at this facility by the number of radiation sources in blister packs. The table shows the average cumulative detection rate for each number of packaged radiation sources. Furthermore, the guidelines recommend a 5% intervention threshold for batch assays. Therefore, we have also indicated the value corresponding to 5% of the average cumulative detection rate, as well as the control range. From this, it can be seen that the control range varies depending on the number of packaged sources. Furthermore, by dividing the average cumulative detection rate by the number of packaged sources, we calculated the detection rate per source. The results show 102.5% for a single source, 46.0% for two sources, 29.2% for three sources, 21.6% for four sources, 17.0% for five sources, and 3.3% for 20 sources. This demonstrates that, as the number of packaged sources increases, the detection rate per source decreases in theory.

Number of packed radiation sources ※1	1	2	3	4	5	20
Detection efficiency of mean value ※2	102.5	92.1	87.5	86.4	85.1	67.0
5% of mean value (tolerance)	5.1	4.6	4.4	4.3	4.3	3.3
Mean value +5%	107.7	96.7	91.9	90.7	89.4	70.3
Mean value -5%	97.4	87.4	83.1	82.0	80.9	63.6
Detection rate per radiation source ※2/※1	102.5	46.0	29.2	21.6	17.0	3.3

**Figure 7:**
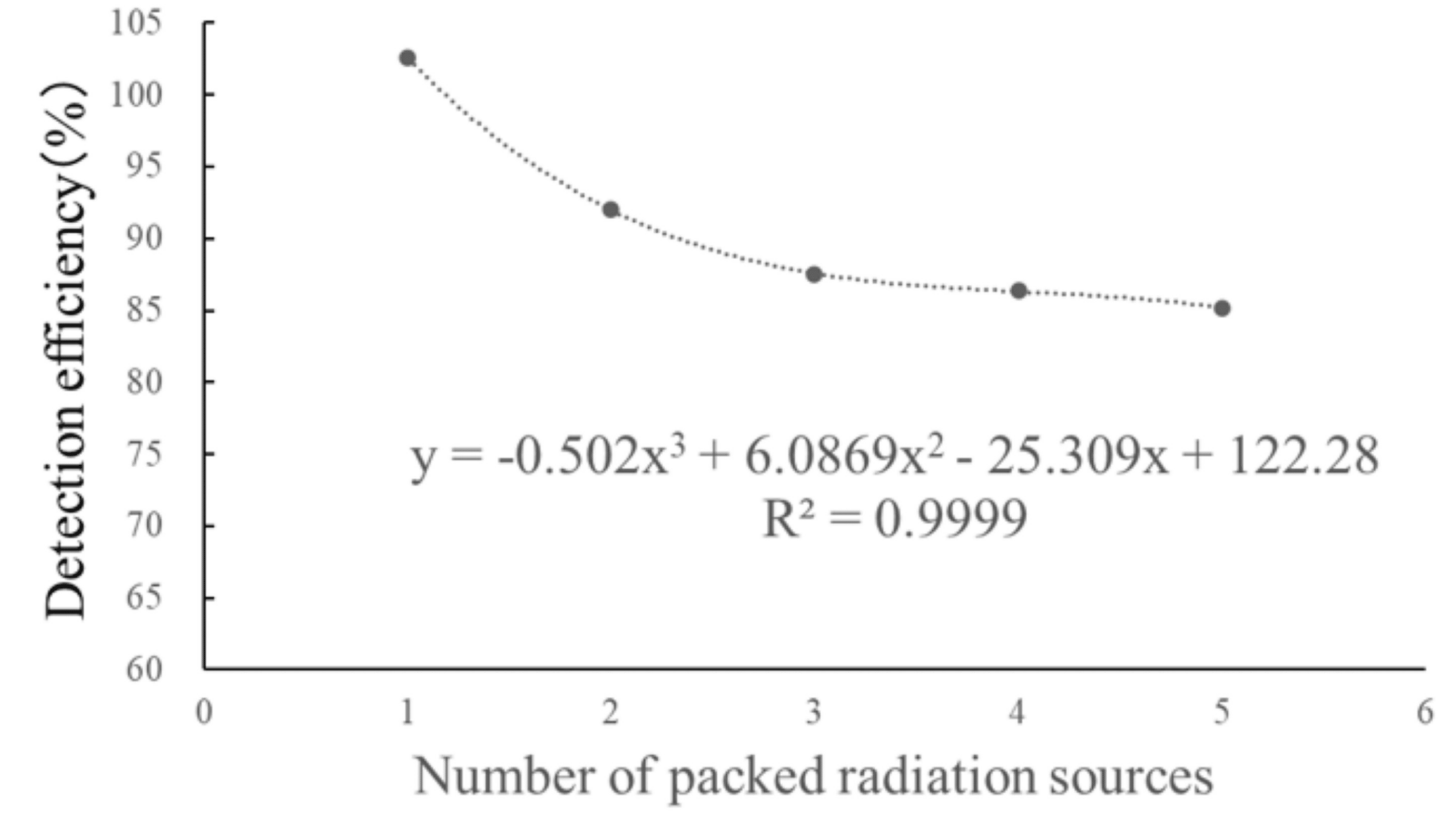
Regression formula for detection rate of one to five packing radiation sources.

The regression equation for one to five packaged sources is as follows: $y=-0.502x^{3}+6.0869x^{2}-25.309x+122.28 (R^{2}=0.9999)$. As shown in Figure 7, a third-order polynomial exhibited a good correlation.

**Figure 8:**
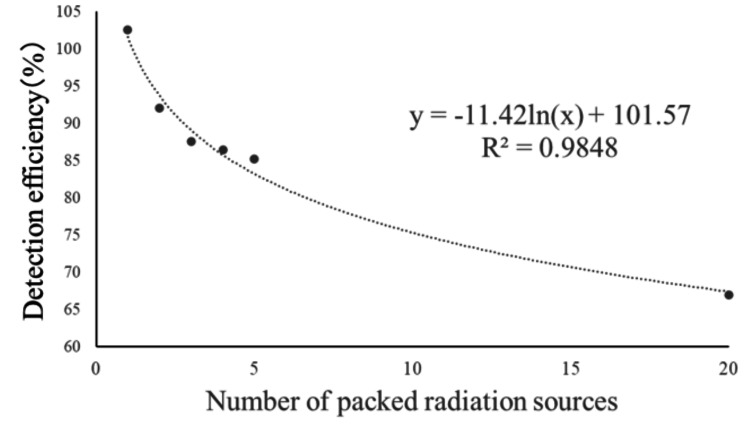
Regression formula for detection rate of one to 20 packing radiation sources.

The regression equation for the number of packaged radiation sources when it is between 1 and 20 is as follows: $y=-11.42ln(x)+101.57 (R^{2}=0.9848)$. As shown in Figure 8, the logarithmic approximation demonstrated better correlation than the polynomial approximation.

## Discussion

In regression models with one to five packaged radiation sources, the detection rate decreased as the number of packaged radiation sources increased. The average detection rate for a single source was 102.5%. Furthermore, compared to the decrease in detection rate when the number of packaged radiation sources increased from one to two, the difference in detection rate decreased as the number increased from two to three and from three to four. In the regression equation for one to 20 packaged sources, when the equation for one to five sources was extended to 20 sources, the data did not plot on the regression line, indicating a deviation. Regarding factors affecting the detection rate, individual variations in source intensity per blister pack were considered. Although the tolerance for the intensity of each source is ±6%, it is unclear whether any of the sources verified by the single-seed assay provided by the supplier upon delivery are included in the package. Therefore, it is possible that all sources within the cartridge are close to the upper tolerance limit of +6%, or the opposite may be true. Additionally, shielding caused by adjacent sources was considered a factor. The results show that the detection rate decreases as the number of sources in a blister pack increases. As can be seen from the X-ray fluoroscopy images, this is likely due to the fact that adjacent sources are densely arranged within the cartridge, causing some of the radiation to be attenuated by the materials of the adjacent sources, such as titanium, silver, and iodine. It was inferred that this shielding effect acts to reduce the detection rate. Furthermore, upon reviewing the X-ray fluoroscopic images acquired to date, it was found that not all products had sources uniformly arranged within the cartridge. Specifically, it was determined that some products had slightly uneven spacing between sources, while others were not arranged in a straight line but were offset to the left or right.

**Figure 9:**
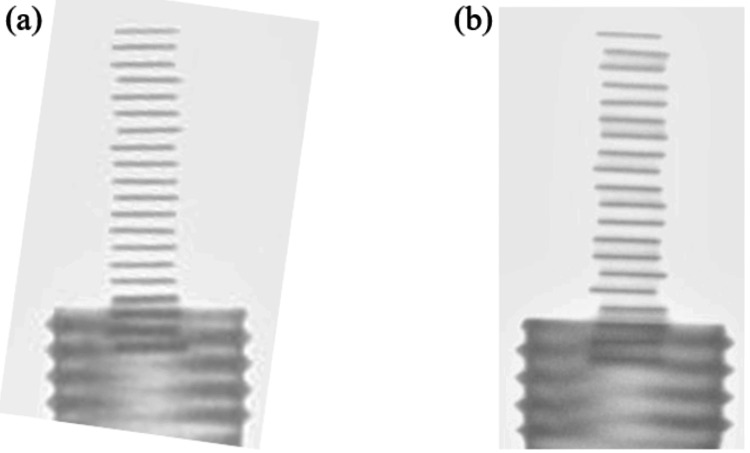
Differences in source alignments by product. Compared to (a), the light sources in (b) are offset to the left and right; however, this difference cannot be detected by visual inspection. Both products functioned without any clinical issues.

Figures 9a, 9b illustrate the differences in the arrangement of light sources for each product. Figure 9a shows all light sources arranged at equal intervals without any lateral offset. As shown in Figure 9b, there were cases where the arrangement was somewhat irregular, indicating that there is some variation from product to product. It was inferred that the arrangement of the radiation sources also affects the shielding mentioned earlier and serves as a factor causing fluctuations in the detection rate. Regarding the impact of shielding on the detection rate, it was considered that the aforementioned complex factors are at play.

Regarding the effect of the stainless steel shielding inside the cartridge, as shown in Figures 4a-4c, approximately three out of every 20 blister packs are sterilized while covered by the stainless steel. It is believed that this stainless steel shielding blocks the majority of the photons. When the 20-pack blister packs were included in the baseline data, it was concluded that this shielding effect was a major factor in why the results for cartridges containing one to five units did not fit the regression equation. If a dead seed is present inside the stainless steel, it may be possible to determine this by the presence or absence of silver wires; however, verifying that the source intensity is correct remains a future challenge.

Based on these results, we identified the product characteristics of AgX100 and derived two regression equations corresponding to the number of packaged sources. As mentioned earlier, the regression equation that includes 20-pack blister packs is likely affected by the shielding effect of the stainless steel, compared with the equation for one to five packs. When conducting verification using batch assays, it is necessary to set a control limit in accordance with guidelines based on the cumulative detection rate accumulated at the facility. The guidelines recommend reporting to the manufacturer if the measured source intensity differs by 5% or more from the nominal value. At our facility, none of the blister packs delivered during the target period deviated from the control limit. Taking the five-seed blister pack as an example, given that the average detection rate for all blister packs delivered to date is 85.1%, a ±5% tolerance (intervention value) corresponds to 4.3%. Therefore, it was considered that, as long as the detection rate falls within the range of 80.9-89.4%, the source strength meets the nominal value. Figures 7, 8 show the trends in detection rates for the five-unit and 20-unit blister packs, respectively. Although there is some variation within the tolerance limits, it can be seen that no blister packs deviate from the tolerance limits. Naturally, as shown in Table 2, the control range for the detection rate corresponding to ±5% varies depending on the number of sources packed in the blister pack.

Regarding detection capability in the presence of dead seeds, it is critically important for the source intensity to meet the specified nominal value during treatment. If even a single dead seed is present in a pack of five blister packs, a 17.0% reduction in the detection rate is expected; since this exceeds the intervention threshold, it can be reliably detected. However, as the number of sources per blister pack increases, the proportion of the detection rate attributable to each individual source decreases, suggesting that the difficulty of detecting a dead seed will increase. Theoretically, if there is one dead seed in a pack of 20 blister packs, the reduction in detection rate would be equivalent to the intervention threshold. Therefore, assuming a dead seed is inside the stainless steel, it is inferred that the detection rate would decrease further due to shielding by the stainless steel. Based on the above, even if a dead seed were mixed into a pack of 20 blister packs, if the pack contained a large number of products with a source intensity of approximately +6% relative to other sources, it is possible that the source intensities would cancel each other out, resulting in a reading within the intervention value. However, the presence of silver wires can be confirmed using X-ray fluoroscopy, and it is considered possible to determine whether they have a source intensity. Based on the above, it is considered that, limited to blister packs of 20, the presence or absence of dead seeds cannot be reliably verified without performing a single-seed assay.

Regarding detection capability when sources with different strengths are present, ensuring that each individual source has the intended strength is critical to the success of 125I seed brachytherapy. However, as discussed earlier, it is difficult to rigorously evaluate source strength without unsealing the source. Therefore, we have no choice but to rely on the single-seed assay data provided by the manufacturer. That said, in accordance with guidelines, users should perform as much verification as possible using dosimeters and other equipment available at their facility. The regression equation derived in this study serves as one alternative method for verifying source strength without breaking the sterility seal. While challenges remain regarding the detection of source strength in blister packs containing 20 seeds, establishing an appropriate tolerance range for source strength verification at our facility has contributed to safer treatment. We have listed several potential limitations below.

When ordering radiation sources, blister packs of five or 20 units are most common. Furthermore, for quantities ranging from one to four units, we order no more than one blister pack per case. Consequently, the sample size for measurement data regarding blister packs containing one to four units is small. We intend to continue accumulating data and will report on this again once we have collected a larger volume of measurement data. The regression equation derived in this study is based on detection rates obtained using specific sources and dosimeters employed at our facility. Consequently, if the type of source or dosimeter differs, the resulting detection rates will naturally vary, and deviations from the regression equation are possible.

The AgX100 cartridges cannot be re-sterilized, and single-seed assays are not possible unless additional cartridges are ordered. In the AgX100, part of the radiation source inside the cartridge is shielded by stainless steel. Consequently, in a pack of 20 blisters, the approximately three radiation sources, each covered by stainless steel, make it difficult to achieve highly accurate measurements.

## Conclusions

In Japan, 125I seed brachytherapy was introduced in 2003 and has since been used to treat many patients. One of the advantages of 125I seed brachytherapy is the low incidence of reported adverse events. If the activity of the 125I source differs from the manufacturer’s nominal value, it may affect treatment outcomes. Guidelines in Europe and the United States state that it is the responsibility of the medical physicist to verify source activity before treating patients. Previous studies have revealed that in Japan, the number of facilities performing source verification is limited due to inadequate verification equipment, a lack of knowledge regarding verification procedures, a shortage of personnel to perform verification, and the inability to re-sterilize many of the sources distributed domestically. If source verification is neglected, the prescribed tumor dose may not be achieved and may also lead to radiation-related adverse events such as urinary retention and rectal fistula.

In this study, we calculated the source-intensity detection rate as the ratio of the source intensity measured with a well-type ionization chamber to the calculated value, and derived a regression equation relating this ratio to the number of packaged sources. Taking into account the shielding effect of the stainless steel components, we derived regression equations showing the best correlation for cases with and without 20 blister packs. A polynomial equation showed good correlation when 20 blister packs were not included, while a logarithmic equation showed good correlation when they were included. As stated in the guidelines, source verification is essential for performing high-quality 125I seed brachytherapy. The official guidelines of the Japanese Society for Radiation Oncology have been published, recommending source-count verification and source-intensity measurement as part of source quality control. We believe the regression equations derived in this study will serve as a reference for facilities that have already started 125I seed brachytherapy, as well as those planning to do so in the future. Understanding the characteristics of acquired measurement data and applying the results to clinical practice is an important responsibility of medical physicists responsible for quality assurance.
